# Quantitative imaging for radiotherapy purposes

**DOI:** 10.1016/j.radonc.2020.01.026

**Published:** 2020-05

**Authors:** Oliver J. Gurney-Champion, Faisal Mahmood, Marcel van Schie, Robert Julian, Ben George, Marielle E.P. Philippens, Uulke A. van der Heide, Daniela Thorwarth, Kathrine R. Redalen

**Affiliations:** a Joint Department of Physics, The Institute of Cancer Research and The Royal Marsden NHS Foundation Trust, London, United Kingdom; b Department of Oncology, Odense University Hospital, Denmark; c Department of Clinical Research, University of Southern Denmark, Odense, Denmark; d Department of Radiation Oncology, the Netherlands Cancer Institute, Amsterdam, The Netherlands; e Department of Radiotherapy Physics, Royal Surrey NHS Foundation Trust, Guildford, United Kingdom; f Radiation Therapy Medical Physics Group, CRUK/MRC Oxford Institute for Radiation Oncology, University of Oxford, United Kingdom; g Department of Radiotherapy, University Medical Center Utrecht, The Netherlands; h Section for Biomedical Physics, Department of Radiation Oncology, Eberhard Karls University of Tübingen, Germany; i Department of Physics, Norwegian University of Science and Technology, Trondheim, Norway

**Keywords:** ADC, apparent diffusion coefficient, DCE, dynamic contrast-enhanced, DW-MRI, diffusion-weighted MRI, FDG, fluorodeoxyglucose, GTV, gross tumour volume, ROI, region of interest, SNR, signal-to-noise ratio, STARD, standards for reporting of diagnostic accuracy, SUV, standard uptake value, QA, quality assurance, QIB, quantitative imaging biomarker, QIBA, quantitative imaging biomarker alliance, Biomarkers, Tumor, Multiparametric magnetic resonance imaging, Positron-emission tomography, Radiotherapy, Multimodal imaging, Review

## Abstract

Quantitative imaging biomarkers show great potential for use in radiotherapy. Quantitative images based on microscopic tissue properties and tissue function can be used to improve contouring of the radiotherapy targets. Furthermore, quantitative imaging biomarkers might be used to predict treatment response for several treatment regimens and hence be used as a tool for treatment stratification, either to determine which treatment modality is most promising or to determine patient-specific radiation dose. Finally, patient-specific radiation doses can be further tailored to a tissue/voxel specific radiation dose when quantitative imaging is used for dose painting. In this review, published standards, guidelines and recommendations on quantitative imaging assessment using CT, PET and MRI are discussed. Furthermore, critical issues regarding the use of quantitative imaging for radiation oncology purposes and resultant pending research topics are identified.

## Highlights

• Quantitative imaging (QI) is promising for radiotherapy.
• The key points from the QI track of the 2nd ESTRO Physics Workshop are discussed.
• QI biomarkers may be used to assess the state of tumours throughout treatment.
• Next steps for using QI in daily radiotherapy routine are identified.
• QI biomarkers are mainly studied in exploratory studies; larger studies are desired.

## Introduction

From treatment planning to response monitoring, imaging is central to ensure optimal outcome from radiotherapy treatment [1]. However, in radiotherapy, most imaging routines assess relative image signals merely to determine the location and size of tumours. Utilizing the full potential of modern imaging also includes assessing tissue function and biological state using quantitative imaging biomarkers (QIBs) [2]. The QIB alliance (QIBA) defines quantitative imaging as “the extraction of quantifiable features from medical images for the assessment of normal or the severity, degree of change, or status of a disease, injury, or chronic condition relative to normal” and QIB as “an objective characteristic derived from an *in vivo* image measured on a ratio or interval scale as an indicator of normal biological processes, pathogenic processes, or a response to a therapeutic intervention” [2].

Quantitative imaging can play a major role in radiotherapy, where tissue sensitivity to treatment is related to microscopic processes, such as metabolism (i.e. as measured by PET, MRI), hypoxia (PET, MRI), perfusion (CT, MRI) and diffusivity (MRI). There are three main areas where quantitative imaging may be used in radiotherapy. Firstly, for more accurate delineation of target volumes in treatment planning, as quantitative images often have a good tumour-to-background contrast [3], [4]. Secondly, QIBs may be used for response monitoring and treatment stratification, by deciding the optimal treatment modality and the optimal dose [5], [6], [7], [8]. Thirdly, quantitative imaging could be used for dose painting, with the radiation dose spatially redistributed throughout the target volume depending on the quantitative parameter maps [9], [10]. Despite these three promising applications, currently quantitative imaging is mainly used as a research tool, primarily for response monitoring with only very limited studies on implementing dose painting and no studies relating to dose stratification. Hence, the full clinical potential of quantitative imaging remains unexploited.

**Fig. 1:**
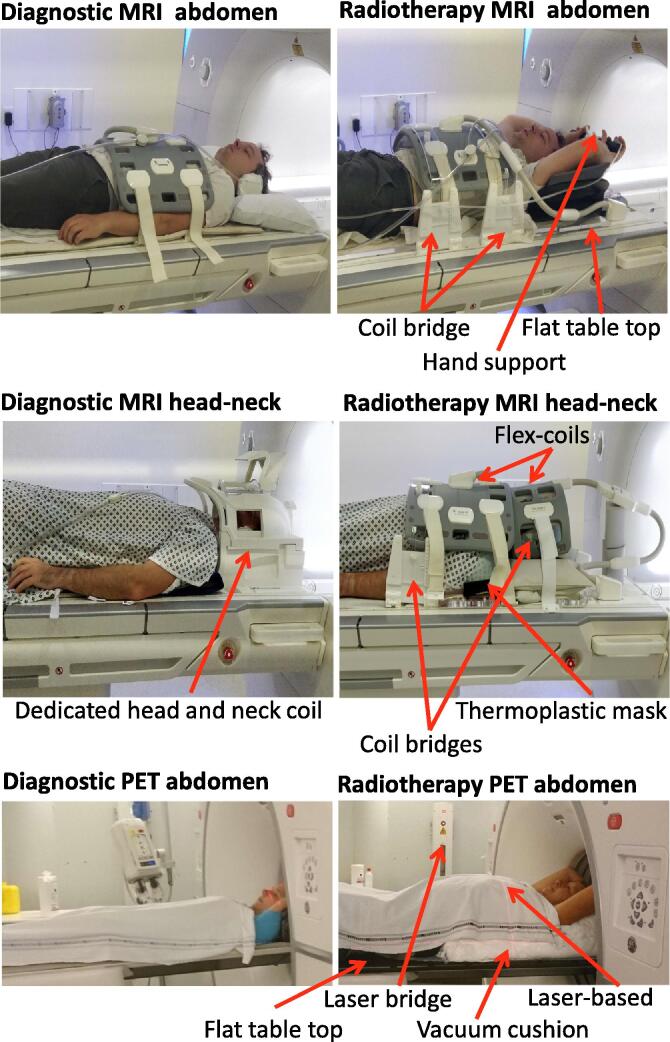
Diagnostic (left) and radiotherapy (right) setup for different modalities (MRI top two rows and PET bottom row) for different indications (from top to bottom: abdomen, head and neck, abdomen).

Despite quantitative imaging for radiotherapy applications being investigated since the early 1990s [11], significant hurdles still need to be overcome to reach widespread clinical adoption. From a technical point of view, different vendors, machines and imaging protocols provide quantitatively inconsistent images. Furthermore, quantitative imaging for radiotherapy is not a direct transposition of quantitative imaging for radiology. For example, the patient positioning in scans for radiotherapy needs to be as similar as possible to the treatment setup (examples shown in Fig. 1) so that the images can be used for treatment planning [12]. For PET, the immobilisation equipment and positioning aids can cause additional attenuation of the signal, whereas in MRI image quality can be affected substantially [13].

In summary, quantitative imaging holds great potential for radiotherapy purposes but is not currently used routinely. At the 2nd Physics Workshop of the ESTRO in 2018, we therefore set out to identify the hurdles that need addressing for quantitative imaging to make the transition from research to clinical use in radiotherapy. The authors and acknowledged members of this article were the organisers and participants of the “quantitative imaging” track at this workshop.

This paper reports on the key points discussed at this meeting and identifies the next steps for quantitative imaging to be used in daily routine in a radiotherapy setting. We have limited this review to the traditional quantitative imaging parameters (sometimes referred to as functional imaging), meaning that high-dimensional features/radiomics are not discussed.

## Quantitative imaging approaches

There are three approaches to acquiring quantitative images. The most straightforward approach is where the signal obtained on the scanner directly relates to a tissue-specific quantity (e.g. Hounsfield units in conventional CT) and hence the obtained image immediately represents a spatial distribution map of this parameter, called a quantitative parameter map. The second type (e.g. conventional PET) is where a single acquired image needs normalization using external parameters (i.e. dosage, body weight, blood haematocrit values) to generate the quantitative parameter map. The third type (e.g. dynamic contrast-enhanced MRI/CT) requires the acquisition of multiple images. Per voxel, a model (e.g. a tracer kinetic model) is then fitted to obtain one or multiple quantitative parameters (e.g. blood volume fraction and blood flow). Most quantitative imaging approaches are pushing the imaging systems to their extremes and often result in considerably larger voxels (e.g. PET typically achieves 5 × 5 × 5 mm^3^, quantitative MRI typically 2.5 × 2.5 × 5 mm^3^) and worse signal-to-noise-ratio (SNR) when compared to what is conventionally used in the clinic, such as CT imaging (typically 0.5 × 0.5 × 1 mm^3^).

**Fig. 2:**
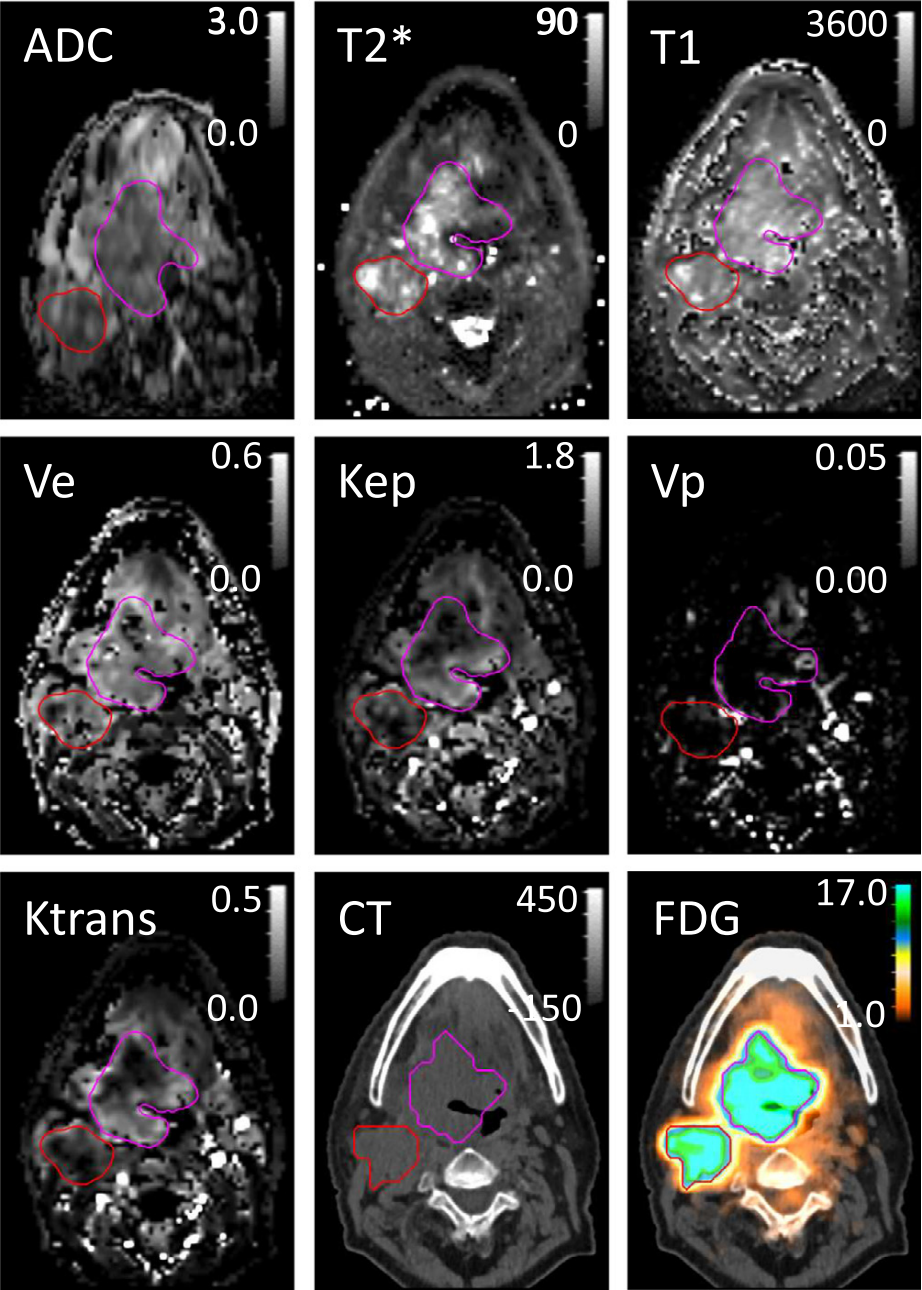
Examples of different quantitative images taken from a single patient with head and neck tumour (pink contour) and a metastatic lymph node (red contour). Top row shows diffusion-weighted MRI (apparent diffusion coefficient (ADC [10^−3^ mm^2^/s]) and relaxometry (T2* and T1 time constants [ms]), second and third row show dynamic contrast-enhanced MRI (fractional volumes of extracellular extravascular space V_e_ and plasma Vp, influx mass transfer rates of gadolinium from plasma to the extracellular extravascular space K^trans^ [min^−1^] and reflux rate from the extracellular extravascular space to the plasma K_ep_ [min^−1^]), CT [HU] and PET (18F-fluorodeoxyglucose (FDG) [g/ml]). Three sets of contours of the primary target (pink) and lymph node (red) were drawn, one on the deformed DW-MRI, one on the registered PET/CT and one for the remaining MRI. Images were taken as part of the INSIGHT trial [74], [78]. (For interpretation of the references to colour in this figure legend, the reader is referred to the web version of this article.)

There are many quantitative imaging approaches available. We discuss the most common approaches in radiotherapy: diffusion-weighted MRI (to signify it is an MRI technique it is abbreviated as DW-MRI; also commonly abbreviated as DWI in literature), dynamic contrast-enhanced (DCE) MRI, MRI relaxometry, CT and PET (examples shown in Fig. 2). A more extensive review on the literature of these techniques (and spectroscopy) within a radiotherapy framework is given by Press et al. [14]. Note that despite some quantitative imaging techniques (such as MR or resonant ultrasound spectroscopy; chemical exchange saturation transfer MRI; hyperpolarized MRI; arterial spin-labelling MRI; dynamic susceptibility contrast MRI; MR or ultrasound elastography; and DCE ultra sound) are not explicitly discussed in this chapter, the general findings on quantitative imaging discussed in this paper still hold for such approaches.

## MRI

### Diffusion-weighted MRI

DW-MRI is a popular research tool in radiotherapy planning and response assessment [15], [16]. In DW-MRI, the MRI signal is typically sensitized to diffusion (e.g. random motion) of water molecules within the tissue. The sensitized signal decays as a function of the amount of diffusion-weighting applied, known as b-value. The amount of diffusion occurring in the tissue, predominantly in the extracellular space [17], [18], is quantified as the apparent diffusion coefficient (ADC; Fig. 2) [19]. Quantitative DW-MRI requires images at two or more b-values to obtain the ADC. The interpretation of ADC is complex, but in general, tumour tissue with high cell density will have a low ADC (restricted diffusion) whereas necrotic tissue will have higher ADC (more free diffusion) than their healthy surroundings. An increase in ADC during treatment is often associated with tumour necrosis and, hence, good treatment response. More complex diffusion models, which further probe tissue microstructure [20] and perfusion [21], have shown potential in radiotherapy [22], [23], [24]. However, such models require more images at different b-values, resulting in longer acquisition times. The analysis is also more complex and often in-house developed analysis is required. Koh and Collins give an introduction to DW-MRI in oncology [16] and Tsien et al. and Liebfarth et al. give reviews of DW-MRI for radiotherapy in particular [15], [25]. DW-MRI is prone to artefacts, which are further discussed in detail by le Bihan et al. [26]. When implementing DW-MRI, we encourage readers to follow guidelines, such as those published by the QIBA [27].

### MRI relaxometry

MRI relaxometry refers to quantifying spin relaxation time constants T1, T2 and T2* (Fig. 2c). These time constants describe the relaxation of a net magnetization from a perturbed state back to equilibrium, as described by the Bloch equations [28]. Their values depend on intrinsic biophysical tissue properties, the total magnetic field strength, and, for T2*, the local perturbations therein. Combined with proton density, these relaxation time constants are the basis of most common MRI contrasts. Hence, tumour contrast in conventional non-quantitative MR images, used for instance for contouring, is reflected in these quantitative parameters. Furthermore, T2* has been associated with tumour hypoxia [29], [30], [31], although the technique is challenging due to the high sensitivity to artefacts. Relaxometry is not always straightforward on clinical MRI systems, and often in-house developed sequences and analysis are required.

### Dynamic contrast-enhanced MRI

Another popular method for tumour diagnosis and characterisation is DCE MRI (Fig. 2b). For DCE MRI, patients receive an intravenous injection of contrast agent (often gadolinium-based) during continuous image acquisition. Gadolinium contrast agents decrease the local T1 relaxation time and hence by continuously acquiring T1-weighted images their distribution within the patient can be studied. By modelling the observed dynamics of the contrast agent distributing in tissue, tissue perfusion and capillary permeability can be quantified. Blood vessels in tumours exhibit a disordered structure, with high dilation and permeability, which can be studied with DCE MRI [32]. Indirectly, parameters related to tumour hypoxia can be investigated. There is a trade-off between temporal and spatial resolution of the acquired images and choices are based upon the desired analysis. The analysis of DCE MRI ranges from a qualitative assessment of the contrast distribution to full pharmacokinetic modelling [33], [34], [35]. The former allows for longer scan times per image (~20–90 s), enabling high spatial resolution. For the latter, a high temporal resolution (1–3 s/image) is required, limiting the spatial resolution. Furthermore, a pre-contrast measurement of the T1 relaxation rate is necessary for absolute concentration quantification of the contrast agent. Khalifa et al. published an overview of different DCE MRI models and their requirements [36], and its use in radiotherapy is described by Zahra et al. [37]. Additionally, Matsuo et al. describe perfusion and hypoxia measurements for radiotherapy [38]. When implementing DCE MRI, we encourage readers to follow the radiology guidelines, such as those published by QIBA [39].

### MRI-specific challenges

There are MRI-specific challenges to take into consideration. For radiotherapy planning in general, imaging protocols must be optimized due to a different coil setup and a larger field of view (FOV). For most radiotherapy purposes, the patient setup needs to be as similar to the radiotherapy treatment setup as possible. Hence, setup aids are often used, such as a flat tabletop and positioning devices similar to those in the treatment room (Fig. 1 top two rows). The setup can be uncomfortable (e.g. hard flat tabletop instead of soft curved tabletop; a fixation mask; hands above head), limiting the scanning time compared to diagnostic scanning. To ensure patient contours are not deformed, MR receiver coils are often placed on coil holders, slightly away from the patient (Fig. 1 top two rows) and possibly requiring the use of less sensitive coils with fewer coil elements (Fig. 1, top row) [12]. Reducing the amount of coil elements restricts the amount of image acceleration possible by parallel imaging and, hence, increases scan duration. The use of less sensitive coils and having the coils placed further away from the subject results in lower SNR.

Geometric deformation is another challenge [40]. When deformed images are used to generate treatment plans they may have an effect on treatment efficacy. The three main sources for geometric deformations include scanner-specific gradient non-linearity, main field non-uniformity, and patient-specific magnetic field perturbations. The gradient non-linearity is constant and, depending on location, can largely be accounted for on most commercial scanners.

Specific to quantitative MRI is the patient-specific magnetic field distortions due to echo-planar imaging (EPI) MRI typically used in DW-MRI. These deformations can be substantial (in the order of mm–cm), particularly close to air-tissue boundaries and implants [41], [42]. The relationship between image resolution, SNR and acquisition time should also be recognised [43]. The appropriate trade-off between these parameters depends on the application.

### CT

As the imaging workhorse in radiotherapy planning and evaluation, CT (Fig. 2d) is well placed to maximise the clinical impact of quantitative imaging with minimum disruption in the radiotherapy pathway [44]. CT has been used quantitatively for decades, with the derivation of electron density data being fundamental to dose calculation. However, there is increasing awareness of the largely untapped potential in paying more heed to the precise distribution of electron density information within an image. While local granularity of Hounsfield units has a negligible impact on macroscopic dose calculations, it can provide clues to the underlying tumour biology, potentially indicating tissue radiosensitivity for instance, or helping to demarcate between recurrence and radiation-induced lung injury following radiotherapy [45], [46], [47]. Furthermore, DCE images can be obtained on a CT scanner, typically using iodine as contrast agent [48], [49]. A drawback with DCE CT compared to DCE MRI is the high radiation dose associated with the repeated imaging during DCE CT acquisition. For radiotherapy patients who already are receiving substantial treatment dose, the additional dose may be acceptable.

### PET

In recent years, PET has shown to be of high value for radiotherapy in terms of staging [50] and accurate target volume delineation [51], [52]. In PET, the patient is injected with a radioactive substance emitting positrons. Following a positron–electron annihilation process, the scanner detects the emitted 511 keV photon pairs and, accordingly, quantifies the distribution of the emitter throughout the patient after several signal correction and normalization steps. PET scanners are often integrated with a CT, or (more recently, but still rarely) MRI scanner, to obtain a geometrically matched high-quality reference image of the patient's anatomy, and to generate high-resolution data for attenuation correction of the originally registered coincidence data. PET tracers for imaging different biological processes can be injected. Most commonly, 18F-fluorodeoxyglucose (FDG), a marker for glucose uptake, which is increased in most tumour types, is injected. Quantitative PET information is derived as the standardized uptake value (SUV) (Fig. 2e), which is a normalization of the tracer distribution to patient body weight and the initially injected total tracer activity. SUV of FDG-PET has been shown to correlate to treatment outcome [53]. Quantitative imaging using kinetic analysis of dynamic PET data acquired with dedicated hypoxia tracers (e.g. [18F]-fluoromisonidazole or the 2-nitroimidazole nucleoside analogue [18F]-HX4) have been associated to radiotherapy outcome in head and neck cancer [54], [55]. Hence, quantitative PET is a promising imaging modality for future quantitative image-guided radiotherapy. Sattler et al. have published a review on PET in radiotherapy [56]. We would advise anyone starting with PET to study the different guidelines, such as those from the European Association of Nuclear Medicine [57], [58], [59].

## Applications in radiotherapy

### Contouring

Accurate contouring of target volumes can benefit from quantitative imaging approaches that give high contrast-to-noise ratio between the tumour and the adjacent tissue structures, such as PET [4], [60], DCE MRI [61] and DW-MRI [61]. The raw data images (pre-modelling) can have a higher contrast-to-noise ratio than the derived parameter maps. However, the quantitative parameter maps can lead to more precise and quantitative contouring rules, which potentially are easier to automate. Dirix et al. and Paulson et al. provide overviews on the use of MRI for contouring, including quantitative imaging [40], [62].

There are challenges when using quantitative imaging for contouring. Tumours are often heterogeneous and, as a consequence, they are perceived differently with different quantitative imaging modalities, as well as compared to conventional images (e.g. contours on PET, DW-MRI and DCE in Fig. 2 are different). For example, Dalah et al. [63] show that gross tumour volumes (GTVs) contours on PET were, on average, more than four times larger than when defined on DCE MRI, and GTVs defined on DW-MRI were more than three times larger than when defined on DCE MRI. The established GTV to clinical target volume margins [64], which incorporate the microscopic spread of tumour cells as identified on population-based pathology data and associated patterns of recurrence data, have been optimized for CT and, hence, may be wrong or redundant for quantitative imaging.

Contouring on quantitative images is complex, especially when incorporating information from multiple modalities. Close collaboration with radiology departments is necessary when introducing such imaging modalities, and voxel-wise correlation of quantitative imaging with pathology and treatment response could be helpful.

### Stratification and response monitoring

QIBs can potentially be used for treatment stratification (individual dose determination or treatment modality selection) either using a single baseline value, or by monitoring (early) tumour response using repeated measures and adapting treatment accordingly. For instance, a tumour that is poorly perfused might benefit from surgery, as poor perfusion and hypoxia are associated with a decreased effectiveness of radiotherapy [65]. These QIBs are typically derived at a whole-tumour level (median/mean tumour QIB value), although other approaches, such as radiomics and voxel-wise approaches, are also being explored [66], [67], [68], [69].

For QIBs to be useful for treatment stratification they need to be predictive. QIBs are predictive when they predict the effect of a specific treatment (e.g. different QIB values give different relation between dose and treatment response) [70]. However, most research is focussed on whether potential QIBs are prognostic. QIBs are prognostic when they can predict the likelihood of a clinical event (e.g. overall survival) independent of treatment. Potential QIBs can be prognostic at baseline [71], [72], [73] and in repeated measures [74], [75], [76], [77], [78]. Whether the known prognostic QIBs are predictive needs further investigations.

### Dose painting

In dose painting, a heterogeneous dose distribution is delivered throughout the tumour according to the QIB maps, increasing dose in regions that are perceived as being more radioresistant and vice versa [79]. This can be combined with monitoring and adapting treatment according to the tumour's local response.

For dose painting, an accurate and reproducible per-voxel estimation of predictive QIBs is required for a voxel-wise dose–effect relationship. At present, such predictive QIB maps are poorly studied for two reasons. Firstly, it is hard to establish which voxels from the original tumour did not respond to radiation, and hence, to determine the local quantitative parameters that relate to a poor outcome. Secondly, implementing redistribution of radiation dose to study the predictive nature of a QIB may cause an unjustified dose de-escalation to certain parts of the target volume, making it challenging to study dose–response relations. Therefore, trial design is of key importance to introduce dose painting in the clinic. For example, one could overcome this by comparing homogeneous dose escalation within a dose escalation region to QIB-based dose redistribution of the escalated dose within the same region, thereby not delivering doses below conventional treatment doses [80].

## Research strategies and study design

Although many potential QIBs have been proposed, a low number of them guide clinical decisions [81], [82]. There are lots of underpowered exploratory studies showing potential QIBs for radiotherapy. However, an important factor hampering large-scale implementation of quantitative imaging in radiotherapy is the lack of appropriate validation and translation into multi-centre investigations with sufficient statistical power.

O’Connor et al. [83] describe a general QIB implementation roadmap which consists of three stages separated by two translational gaps. For quantitative imaging in radiotherapy, the first stage consists of developing the quantitative imaging approach and showing its capability as a potential QIB *in vitro*, in animals and in humans. The first translational gap is then filled when the promising potential QIB goes from the preclinical and early clinical stage into the second stage, becoming a reliable medical research tool used to test hypotheses in larger studies. Then, to cross the second translational gap and become a clinical decision-making tool (third stage), the QIB must successfully show appropriate technical performance, ability to relate to an underlying biological feature and a meaningful clinical outcome, and should show a cost-effectiveness benefit over current practice.

Unfortunately, most radiotherapy-specific potential QIBs are in the first stage, with exploratory single-centre studies. Common for such studies is the many quantitative parameters evaluated in a limited number of patients, making them underpowered to demonstrate sufficient performance. Furthermore, such studies often overestimate the true performance of the potential QIB, as optimal settings are determined from the same dataset they are tested on. This includes tweaking of post-processing approaches, region of interest (ROI) definition, and ad-hoc determination of cut-off values using receiver operator characteristic analysis. Such cut-off values are based on the same data they are tested on and hence their performance is overestimated. Studies which use cut-off values determined up-front are desired to assess their true performance.

To prevent false positive results, QIB studies should undergo upfront statistical power estimation the same way as clinical efficacy studies. Given that the studies are well-powered there is also a value in publishing negative results. It will contribute to improving future quantitative imaging studies and a platform for the exchange of such findings should be made accessible for the community.

Unfortunately, larger multi-institutional studies are expensive and require more effort than exploratory studies, and hence are performed less frequently. Therefore, it is important to carefully select which QIB to study. To bring promising QIBs forward from the exploratory phase to multi-centre evaluation, the community must join forces and establish unified procedures. Obvious is the value of a larger cohort where multiple institutions collaborate. However, cheaper alternatives that utilize data from many smaller studies can also help build evidence of a quantitative imaging parameter being a QIB. For example, cut-off values from previous exploratory studies published in literature could be validated in new datasets from other sites to determine their “true” sensitivity and specificity. Such validations might be less optimistic but reflect a more realistic estimate to help increasing evidence regarding whether a parameter is clinically useful and should be further explored.

Central in the QIB roadmap is the need for imaging-histopathology correlations. Such correlations build up the understanding of why the quantitative imaging parameter is a QIB and build evidence to select the QIB for larger trial studies. In radiotherapy, there is a lack of such correlative studies that dissect the underlying biology and its relation to quantitative imaging [84]. Different from many general QIB studies is the potential need for spatial correlations in radiotherapy studies. An example is when a subvolume is identified by quantitative imaging for dose painting or dose escalation; if the histopathology analysis finds that the subvolume is dominated by aggressive, hypoxic tumour cells, this would increase the likelihood of continuing with further studies of the identified potential QIB. Such studies [85], [86], [87] are ideally performed on patients that are referred directly to surgery, without radiotherapy. These studies require close collaborations with the radiotherapy, imaging, surgery and pathology departments to obtain images taken in the radiotherapy setup and correlate them to pathology.

To accelerate the clinical introduction of QIBs, a forum for identifying partners for multi-centre investigations is central and should be established and distributed to the radiotherapy community to improve the quality and outcome of future studies. This forum should also enable the sharing acquired data, trial designs and study results.

## Data processing

Most quantitative imaging approaches depend upon acquiring one or more images and applying a model to the data, which returns the quantitative information. For several quantitative imaging approaches, most notably Hounsfield units on CT, the steps from raw data to quantitative information are well established and occur within the framework of the commercial systems. For others, such as DCE MRI, the methods are not standardised [36], and in-house post-processing is required to obtain the quantitative parameter values. Yet others, including DW-MRI and relaxometry MRI, have some vendor solutions but are often performed using in-house tools due to the lack of transparency and customisation of the vendor's tools. These post-processing steps are often not standardised and care must be taken to ensure reliable and generalizable results.

Where possible, it is encouraged to prioritize well-established post-processing methods over developing in-house toolkits. Using well-established post-processing methods decreases the workload, decreases the chance of errors, and increases the generalizability of your results. When in-house post-processing software is used, publishers should be more proactive in encouraging the sharing of those scripts.

Typically, data processing for quantitative imaging can be broken down into three stages. Firstly, pre-processing converts the raw data into a format in which model-fitting can be done. This stage can include registration of multiple image datasets to a common reference frame, resampling of data onto a more appropriate grid spacing and denoising of data. Secondly, the pre-processed data are modelled and quantitative parameters are estimated. Thirdly, post-processing and data reviewing is carried out, which can include further noise-reduction, smoothing and ROI definition.

### Pre-processing

One common pre-processing step, particularly in abdominal imaging, is image registration [88]. There are two main reasons for image registration in quantitative imaging. Firstly, monomodal registration to align two or more images from the same quantitative imaging series that are taken at different time points (e.g. images from a DCE MRI series [89]). Secondly, registration between different quantitative images to a reference image is desirable when combining different modalities or obtaining multiple parameter maps within a modality [90]; note that this step can alternatively be done as post-processing, if based on the parameter maps, instead. Unfortunately, quantitative images are typically low spatial resolution and do not have a clear contrast for the entire anatomy, making both manual and automatic image registration methods challenging. One approach that should be avoided when applying multimodal registration is to simply overlay ‘the spot on the spot’, e.g. assume that a hot-spot on PET overlaps with a region of low ADC, as modalities are complementary [63], [91]. A more appropriate method is to undertake registration based on reference anatomy visible in both imaging modalities. Clinical judgement is subsequently required to determine whether an ROI nearby is properly aligned. Depending on the situation, either rigid or deformable registration can be desired. Deformable image registration may be able to account for changes in patient setup or deformed anatomy due to day-to-day changes (e.g. stomach filling) and deformable motion (e.g. breathing), however, the registration process is more complex and harder to verify [92]. Furthermore, the propagation of dose accumulation in the presence of deformations is not agreed upon [93].

### Parameter estimation

In quantitative imaging, quantitative image parameters need to be extracted from the acquired data, often done by fitting some model. Parameter estimation can be undertaken either in a voxel-wise or an ROI-wise manner [94]. A voxel-wise approach retains the spatial information but relies on a high SNR per voxel. Alternatively, when quantitative imaging is used for treatment stratification or response assessment, it may be preferable to use ROI-wise parameter fitting. By aggregated data from multiple voxels, the SNR is increased, potentially resulting in a more accurate model fit. A downside of this approach is that information on the heterogeneity of the tumour is lost. Furthermore, if tissue is heterogeneous within the averaging ROI, the models might no longer be adequate to describe the data. In such cases, voxel-wise fits may be preferred, which can be analysed for example as histograms from the ROIs. Contouring and dose painting require voxel-wise fitting as decisions are made on a per-voxel basis.

Noisy MRI data is governed by a Rician distribution, which causes a noise-dependent overestimation in MR signal [95]. This results in an additional SNR-dependent bias term in the QIBs when fitting to noisy data (worse accuracy). This can be particularly challenging in radiotherapy due to the suboptimal (compared to radiology) coil setup required (Fig. 1).

Several quantitative imaging models are nested models. One issue of such a model is that data might be uninformative. For example, in a model with multiple parameters, “blood velocity” may have no meaning in voxels with no “blood volume”. An unanswered question is how to deal with such an absence of parameter fitting results in a radiotherapy framework.

Quantitative images come with uncertainties: there are error bars on the fit parameters; and the relation between the quantitative image to treatment response has uncertainties [96]. There is no unified method of dealing with such uncertainties in radiotherapy planning. However, handling uncertainties has always been at the heart of radiotherapy (e.g. geometric uncertainties) and we might be able to account for such uncertainties in, for instance, dose painting approaches.

### Data reviewing

Quantitative imaging requires the processing of large amounts of data. To make use of data from multiple modalities, these data need to be stored and communicated appropriately [56], which is often achieved by DICOM files [97]. The use of a standardised data interchange format enables various options for the use of quantitative imaging, such as carrying out the definition of radiotherapy target volumes on a nuclear medicine workstation and transferring the data via DICOM to the treatment planning system. However, not all treatment planning systems properly represent quantitative imaging data; for example low sub-zero voxel values (ADC has values in the order of 10^−3^ in DW-MRI) are represented as zeros in some systems. Furthermore, some systems have trouble with data that was not acquired axially.

To increase the reproducibility of studies and to enable multi-centre, multi-vendor validation studies, it is desirable to use well-established, freely available data processing tools. An example of such a freely available tool is 3D Slicer [98].

## Standardisation and quality assurance

To progress clinical deployment of quantitative imaging for radiotherapy, we need to ensure that results are transferable between clinical sites. This requires standardisation and quality assurance (QA).

Quantitative imaging should be an effective approach to standardization as it measures quantifiable features on a ratio or interval scale that should not depend on acquisition settings. In theory, it is not necessary to have identical acquisitions as long as the measures (e.g. quantitative parameter maps) are accurate (no bias) and precise (minimal variance). Therefore, for quantitative imaging, standardisation of the QA should be sufficient to guarantee transferable results. However, quantitative imaging models are often simplifications of the real processes going on, and acquisition parameters (e.g. the duration of diffusion weighting gradient) can have unexpected effects on the data that are not explained by the simplified model. As a result, it is important to be aware of which parameters might influence the modelling.

One important tool in QA is phantom measurements. Both commercial and in-house phantoms are developed for quantitative imaging for radiological purposes (e.g. [99]) and these should be adopted by the radiotherapy community. Although phantoms are becoming more complex and tissue-like [100], [101], in practice, phantoms do not cover all possible aspects of variation. For instance, DW-MRI [102] *in vivo* is affected by choice of b-value [21], gradient shape and duration [103], [104], echo time, and inversion time [105], which are not all reflected in any DW-MRI phantoms. Hence, caution needs to be taken and standardisation of imaging protocols is desired.

Many initiatives are working on standardization and QA of quantitative imaging [57], [58], [106], [107]. For many aspects of quantitative imaging and standardisation, the radiotherapy community can build upon the guidelines from these initiatives.

However, contouring and dose painting are considerably different from regular radiology and we need to develop some additional QA for these purposes. For example, QA routines assessing voxel-wise accuracy and precision should be developed. Furthermore, there might be stricter requirements on geometric accuracy than for radiology. In particular, when an MR-only workflow is being considered it is advisable to include QA on B0-heterogeneity and gradient non-linearity in these cases.

## Reporting

Incomplete reporting has been identified as a major reason for waste in biomedical research [108]. This is also strongly the case in quantitative imaging, where methods are often far from standard. Regularly, quantitative imaging studies lack essential information to be repeated; incomplete methodological description, and lack of reporting standards or awareness of these being the most important reasons. One guideline that has been developed to prevent incomplete reporting is the standards for reporting of diagnostic accuracy (STARD) [108]. STARD lists thirty specific items that should be included in every report of diagnostic accuracy study. There are some quantitative imaging specific guidelines from the radiology community on what needs reporting, for example for DW-MRI [106]. Here we propose additional items specific to quantitative imaging in radiotherapy.

The SNR is an important quantity to report, especially in MRI. SNR and contrast-to-noise ratio of the quantitative parameters determines how well subregions can be determined for contouring or dose painting. For MRI, the SNR of the raw data is important as it can cause bias. Determination of SNR can be non-trivial in MRI and the process is described in detail by Goerner et al. [109].

Scanner variation is another source of reduced reproducibility. To ensure the translatability of results between sites, it is desired that the sequence performance (bias and variance) on a standard phantom are reported in publications. For example, Kooreman et al. used several commercial phantoms to characterize the performance of several MR-Linac scanners for quantitative imaging [110]. The phantoms used for this purpose should either be commercial or well documented, such that other sites can easily test whether their scanners have similar performance in this well-defined environment before building upon the published results.

A test–retest study design is encouraged and should be reported along with the result on potential QIBs. A test–retest reliability (also known as repeatability) study assesses whether a potential QIB yields the same result when acquired multiple times (conditions should not have changed) [111]. This analysis allows distinguishing between a real change and a change due to day-to-day random variations or random errors.

Other generally desirable reporting items may count patient immobilization, coil setup, timing of scans, treatment details and patient clinical baseline data. They can all have an impact on the quality of imaging, and cause differences in derived quantities. Inclusion of these details may seem irrelevant for the particular study, but it can be critical information in a meta-analysis or for reproducibility purposes.

Data and algorithm sharing should ideally be a natural part of the scientific method. Some scientific journals require authors to share raw data, source code etc. necessary to reproduce or develop published research. Some funding bodies also require open-access data sharing. Data sharing can be done as supplemental materials to the article, or, ideally, in an indexed repository [112]. We encourage authors to share their data (whilst respecting privacy laws) and algorithms, even when not required by funders or editors.

Finally, some information may be inaccessible and therefore cannot be reported. Imaging attributes are stored in the file header portion as File Meta Information and are accessible through workstations or DICOM readers. Other information, however, is hidden in the header preamble by the manufacturer for proprietary reasons. Manufacturers are urged to more transparency (fewer black boxes). Not having access to the raw data may also limit the understanding of the actual acquired data.

## Future focus

Quantitative imaging has great potential in radiotherapy, and we have outlined the current state and emphasized the associated challenges in this field. Despite its potential, quantitative imaging is still far from being used routinely in clinical radiotherapy. Here we will discuss how to focus research in quantitative imaging to facilitate a clinical introduction in radiotherapy.

Most potential QIBs are studied in an exploratory setting with small sample sizes at single institutions. Introducing quantitative imaging into the clinical workflow requires proving its clinical benefit in large multicentre studies. The radiotherapy community will need to identify which potential QIBs should be investigated in such large studies and setup larger, standardised multicentre studies.

The large variety of implementations of a given quantitative imaging method between sites, studies and vendors is hindering proper investigations. However, fixing imaging and processing parameters can hinder the development of quantitative imaging techniques. The radiotherapy and radiology communities need to come up with ways to allow development while being able to combine quantitative imaging data from different sites.

We encourage the development of radiotherapy-specific imaging solutions, such as dedicated MRI coils, to enable imaging in the radiotherapy setup with image quality similar to diagnostic imaging. Additionally, radiotherapy treatment planning software should become more compatible with quantitative imaging.

In conclusion, quantitative imaging shows great potential for increasing the efficacy of radiotherapy. Quantitative imaging could improve contouring with better tumour visibility. Quantitative imaging might be used to assess the state of tumours at baseline and repeatedly throughout treatment, allowing individual adjustments of the radiation dose. This could increase the efficacy of modern radiotherapy, for which dose is currently population-based, by improving tumour response while minimizing side effects. Finally, local quantitative information could be utilized to take into account the tumour heterogeneity and determine optimal radiation doses for dose painting. We believe it is worthwhile overcoming the challenges discussed and further investigating the added value of quantitative imaging in radiotherapy.

## Conflicts of interest

None.
